# Evaluation of Inflammatory Status in Chronic Kidney Disease Patients and a Comparison Between Hemodialysis and Peritoneal Dialysis Patients

**DOI:** 10.7759/cureus.69443

**Published:** 2024-09-15

**Authors:** Akhil Sahib, Cankatika Choudhury, Imtiyaz A Wani, Muzafar M Wani

**Affiliations:** 1 Neurology, Govind Ballabh Pant Institute of Postgraduate Medical Education and Research, New Delhi, IND; 2 Nephrology, Sher-i-Kashmir Institute of Medical Sciences, Srinagar, IND

**Keywords:** chronic kidney disease (ckd), continous ambulatory peritoneal dialysis, continuous ambulatory peritoneal dialysis, high-sensitivity c-reactive protein (hs-crp), inflammation, interleukin (il)-6, maintenance hemodialysis

## Abstract

Introduction

Inflammation is a common denominator in patients with chronic kidney disease (CKD), which is not explained by the pre-morbid risk factors and can lead to atherosclerosis and increased cardiovascular mortality. We have compared inflammatory markers like high-sensitive C-reactive protein (hs-CRP) and interleukin-6 (IL-6) in patients with CKD undergoing continuous ambulatory peritoneal dialysis (CAPD) and hemodialysis (HD) in both pre-dialysis phase at baseline and post-dialysis phase at three months to determine which dialysis has a more inflammatory burden on the patient.

Materials and methods

Fifty CKD patients diagnosed with end-stage renal disease (ESRD) who fulfilled eligibility criteria were recruited in this prospective observational study over two years. We measured levels of IL-6, hs-CRP, serum albumin, and body mass index (BMI) in both pre-dialysis and post-dialysis phases. Our results were obtained by analyzing 50 patients of ESRD who were planned for renal replacement therapy in the form of bridge dialysis, out of which 25 were put on HD and CAPD each. Pre-dialysis assessment was done at baseline and post-dialysis at three months. Each group served as its own control. IL-6 and hs-CRP levels were performed using commercially available kits employing the enzyme-linked immunosorbent assay (ELISA) method.

Results

Inflammatory markers (hs-CRP, IL-6) were elevated in both the pre-dialysis and post-dialysis phases of CKD, regardless of the dialysis modality. hs-CRP was significantly elevated in the HD group as compared to the CAPD group; however, the difference in IL-6 between the two groups was not significant. Albumin and BMI were significantly low in both groups from baseline, with patients on HD exhibiting lower albumin than the CAPD group, although the result was insignificant.

Conclusion

Markers of inflammation, like hs-CRP and low albumin, are more pronounced in HD than in CAPD. We conclude that the inflammatory burden is higher in patients undergoing HD.

## Introduction

Inflammation has an important role in the etiology of atherosclerosis and endothelial dysfunction in chronic kidney disease (CKD) and end-stage renal disease( ESRD) patients [1]. In patients with CKD, inflammation starts even in the early stages, long before the initiation of renal replacement therapy (RRT). It has been scientifically proven that the risk of mortality due to cardiovascular disease (CVD) is increased in ESRD patients, which is not fully explained by conventional risk factors, thereby indicating the role of other inflammatory disease processes [2]. Compared with the general population, serum concentrations of inflammatory markers like high-sensitivity C-reactive protein (hs-CRP) and interleukin-6 (IL-6) are elevated approximately threefold in ESRD patients due to excess production and reduced renal clearance [1]. It has been postulated that uremic patients exhibit a paradoxical immune response as exhibited in autoimmune disorders like systemic lupus erythematosus (SLE). Several mechanisms are involved in stimulating this inflammation in CKD patients, including patient-associated factors, such as underlying comorbidity, uremia, infections, immunologic or genetic factors, as well as factors related to dialysis treatment. The final outcome is elevated concentration of several cytokines primarily due to their decreased renal clearance and increased cytokine release due to accumulated uremic toxins [3] . However minimal studies have been done to demonstrate which type of maintenance dialysis has lesser inflammatory burden. Markers of inflammation like IL-6 and hs-CRP are elevated in ESRD patients irrespective of etiology of CKD [1]. In addition, these inflammatory markers are shown to be strong prognosticators of poor outcomes. CRP is not only the single most used and extensively studied inflammatory biomarker, but it appears to be a direct risk factor itself and an active promoter of proatherosclerotic process. Increased CRP levels have been associated with elevated mortality in hemodialysis (HD) [4,5] and continuous ambulatory peritoneal dialysis (CAPD) patients [6]. In view of the strong association between CRP and IL-6, it is not surprising that IL-6 is also recognized as a major mediator of the acute phase response. Elevated IL-6 have also been indicated to forecast myocardial infarction and predict mortality in both pre-dialysis and HD patients [7,8] and can be a better predictor of cardiovascular mortality than CRP in these patients [9].Therefore, IL-6 measurement may have clinical utility as a predictive marker for atherosclerosis and its associated comorbidity. In our study, we aimed to evaluate markers of inflammation in patients with ESRD undergoing CAPD and HD to determine the effect of dialysis modality on inflammation. 

## Materials and methods

This was a prospective, single-center, observational study carried out in the Departments of Nephrology and General Medicine at Sher-i-Kashmir Institute of Medical Sciences, Srinagar, India, over two years, from 2013 to 2015. Fifty patients of stage 5 CKD with glomerular filtration rate (GFR) of less than 15 mL/min/1.73 m^2^ with the Modification of Diet in Renal Disease (MDRD) equation and ages between 18 and 70 years were recruited in the study. All these patients were listed for renal transplantation in our institute if found eligible.

The exclusion criteria included patients who refused consent, patients who were already on maintenance dialysis, patients with acute decompensation of their CKD, patients with evidence of active infections, patients on antibiotics within two weeks or recent hospitalization in two weeks, patients with any comorbid disease affecting survival including chronic infections like tuberculosis, malignancies, or autoimmune disorders, patients positive for human immunodeficiency virus, patients on immunosuppression, patients with active liver disease, and patients who had recent vascular events (up to three months of recruitment). The local institutional ethics committee of the hospital approved the study, and written informed consent was taken from all participants. The study complied with the Declaration of Helsinki. 

Fifty patients having ESRD who were planned for conservative management in the form of bridging dialysis were recruited for the study. The sample size was calculated based on ESRD prevalence as mentioned in the article by Agarwal et al. [10]. The calculated sample size was 48, and we rounded it to 50. Pre-dialysis assessment was done, and 25 patients were assigned to undergo HD and CAPD each. The type of dialysis was determined independently of the study and according to the treating nephrologist's and the patient's choice. These patients were subjected to hs-CRP and IL-6 levels before starting dialysis and were reassessed three months later. Simultaneously, serum albumin levels and body mass index (BMI) were checked. Polysulfone membranes were used for the HD process. CAPD solutions from Baxter Healthcare were used. The number of dialysis sessions judged clinically in HD patients was three times/week, and in CAPD, 3-4 times per day, mostly with 1.5% dextrose. All participants remained on their initial dialysis modality and had no crossover at follow-up. Patients on HD were followed three times per week for their maintenance dialysis sessions, and patients on CAPD were followed once a month.

Sample collection and biochemical analysis

Following all aseptic precautions, 4 ml venous blood was collected from patients in sterile serum collection tubes. The tubes were centrifugated for five minutes to separate the serum from the clot. The clear serum was aspirated using disposable plastic droppers, poured into 1.5 ml microcentrifuge tubes, and stored at -80°C until tested for IL-6 and hs-CRP levels. All the assays were carried out using commercially available ELISA kits following the manufacturer’s guidelines.

Statistical analysis

The categorical variables were presented in numbers and percentages (%). The quantitative data having normal distribution were presented as the means ± SD and the non-normal distribution as median with 25th and 75th percentiles (interquartile range). The data normality was checked by using the Shapiro-Wilk test. We used nonparametric tests for cases where the data was not normally distributed. The comparison of the quantitative and nonnormally distributed variables was analyzed using the Mann-Whitney Test, and quantitative and normally distributed variables were analyzed using the independent t-test. Paired t-test/Wilcoxon signed-rank test was used for comparison across follow-up. The comparison of the variables that were qualitative in nature was analyzed using the Chi-square test. Fisher's exact test was used if any cell had an expected value of less than 5. The Spearman rank correlation coefficient was used to compare the correlations of post-dialysis albumin, IL-6, and hs-CRP between the two groups. Data entry was done in the MS Excel (Microsoft Corporation, Redmond, Washington, United States) spreadsheet, and the final analysis was done using the IBM SPSS Statistics for Windows, Version 25 (Released 2017; IBM Corp., Armonk, New York, United States). For statistical significance, a p-value of less than 0.05 was considered statistically significant.

## Results

**Figure 1:**
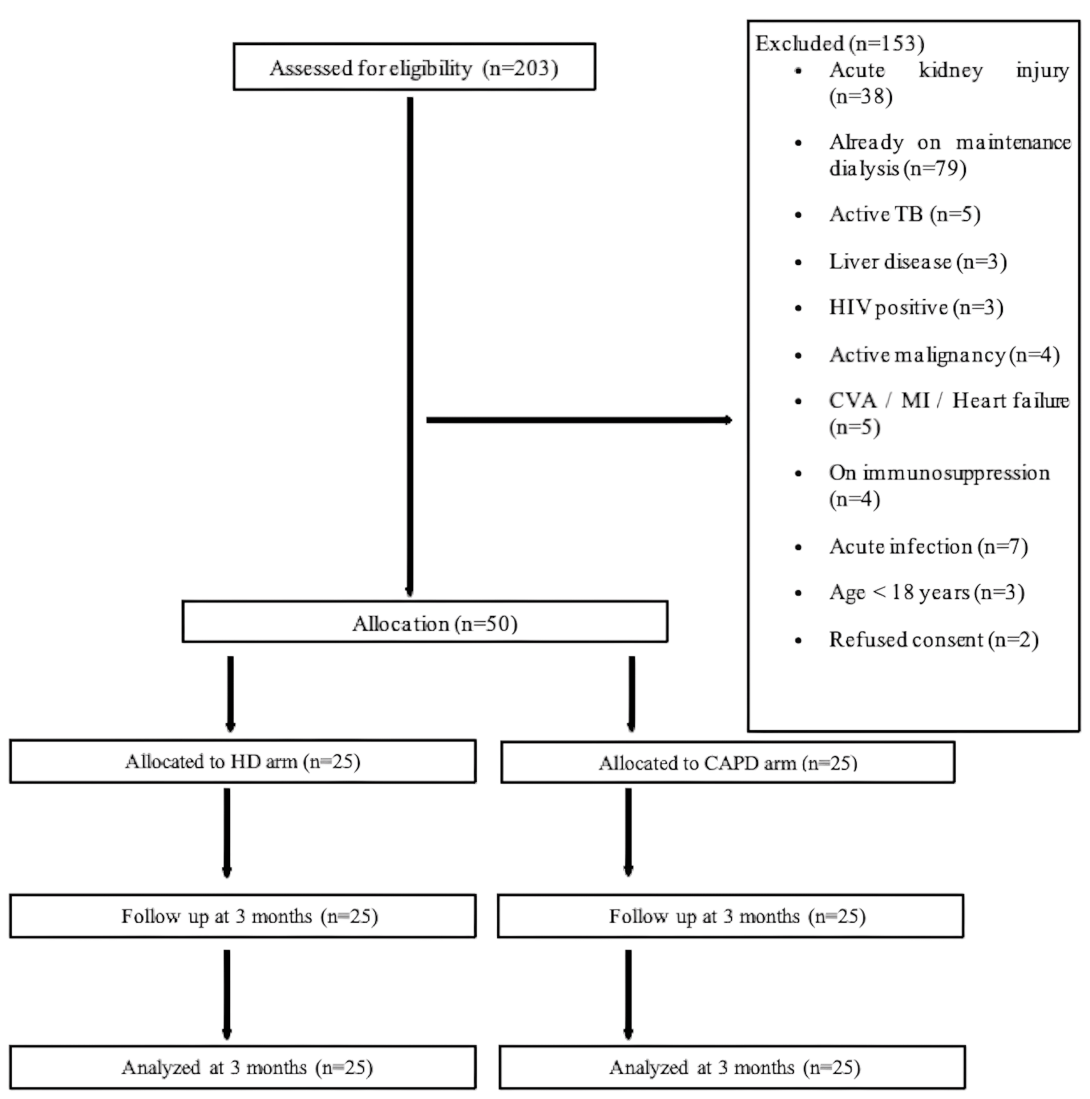
Flow chart of the study HD: Hemodialysis; CAPD: continuous ambulatory peritoneal dialysis; TB: tuberculosis; CVA: cerebrovascular accident; MI: myocardial infarction; HIV: human immunodeficiency virus

A total of 203 patients were screened over a period of one year and nine months for eligibility. Of them, 153 patients were excluded for various reasons (Figure 1). Fifty patients (25 in each group) were recruited for the maintenance bridge dialysis. Baseline inflammatory markers were assessed in all these patients, followed by a repeat sample at three months. No patients were lost to follow-up during the observation phase of three months.

Table 1 outlines the baseline characteristics of both groups. The patients undergoing HD were younger than the patients on CAPD (p-value of 0.018). There was no significant difference between the two groups concerning traditional risk factors like smoking status, hypertension, and dyslipidemia except for diabetes, which was significantly more in the CAPD group (p-value of 0.025). Baseline albumin and BMI were comparable between the two groups, as were the baseline values of urea and creatinine between the two groups (Tables 1-3).

**Table 1:** Comparison of baseline characteristics between CAPD and HD ^‡^Independent t-test; *Fisher's exact test; ^†^Chi-square test CAPD: Continuous ambulatory peritoneal dialysis; HD: hemodialysis; CAD: coronary artery disease; HCV: hepatitis C virus The categorical variables were presented in numbers and percentages (%). The quantitative data with normal distribution is presented as the mean ± SD. A p-value of less than 0.05 was considered statistically significant. The patients undergoing HD were significantly  younger than the patients on CAPD (p-value of 0.018). The percentage of  diabetics was significantly more in the CAPD group (p value of 0.025)

Baseline characteristics	CAPD (n = 25)	HD (n = 25)	Total	p-value
Gender				
Female	6 (24%)	8 (32%)	14 (28%)	1^†^
Male	19 (76%)	17 (68%)	36 (72%)	
Nonsmokers	10 (40%)	14 (56%)	24 (48%)	0.258^†^
Smokers	3 (12%)	0 (0%)	3 (6%)	0.235^*^
Ex-smokers	12 (48%)	12 (48%)	24 (48%)	1^†^
Hypertension	23 (92%)	21 (84%)	44 (88%)	0.667^*^
Diabetes	11 (44%)	3 (12%)	14 (28%)	0.025^*^
Dyslipidemia	3 (12%)	3 (12%)	6 (12%)	1^*^
Heart failure	3 (12%)	0 (0%)	3 (6%)	0.235^*^
CAD in the last 1 year	0 (0%)	0 (0%)	0 (0%)	NA
Stroke	1 (4%)	0 (0%)	1 (2%)	1^*^
Tuberculosis	1 (4%)	0 (0%)	1 (2%)	1^*^
HCV	1 (4%)	0 (0%)	1 (2%)	1^*^
Age (years)	52.64 ± 11.27	45.12 ± 10.51	48.88 ± 11.44	0.018^‡^
Height (cm)	161.64 ± 5.48	163.12 ± 3.8	162.38 ± 4.72	0.272^‡^
Weight (kg)	61.68 ± 8.61	61.72 ± 8.27	61.7 ± 8.35	0.987^‡^
Body mass index (kg/m²)	23.83 ± 3.22	23.01 ± 3	23.42 ± 3.11	0.36^‡^

**Table 2:** Comparison of albumin (g/dL) between CAPD and HD ^‡^Independent t-test; **paired t-test CAPD: Continuous ambulatory peritoneal dialysis; HD: hemodialysis Normal albumin levels: 3.5-5.5 g/dl The quantitative data having normal distribution is presented as the mean ± SD. A p-value of less than 0.05 was considered statistically significant. Serum albumin decreased significantly in both the groups post-dialysis (intragroup p-value of 0.0002 and <0.0001 for CAPD and HD group, respectively)

Albumin (g/dL)	CAPD (n = 25)	HD (n = 25)	Total	p-value
Pre-albumin (g/dL)	3.56 ± 0.56	3.44 ± 0.43	3.5 ± 0.5	0.379^‡^
Post-albumin (g/dL)	3.09 ± 0.25	2.96 ± 0.34	3.03 ± 0.3	0.132^‡^
Intragroup p-value	0.0002^**^	<0.0001^**^	-	-

**Table 3:** Comparison of kidney function test parameters between CAPD and HD ^‡^Independent t-test; ^§^Mann-Whitney U-test; ^¶^Wilcoxon signed-rank test; **paired t-test CAPD: Continuous ambulatory peritoneal dialysis; HD: hemodialysis Normal urea range: 5-20 mg/dl; normal creatinine range: 0.7-1.2 mg/dl The quantitative data having normal distribution is presented as the mean ± SD and the nonnormal distributed data as median with 25th and 75th percentiles (interquartile range). A p-value of less than 0.05 was considered statistically significant

Kidney function test parameters	CAPD (n = 25)	HD (n = 25)	Total	p-value
Pre-urea (mg/dL)	138(89-216)	148(118-203)	141(94-208.75)	0.6^§^
Post-urea (mg/dL)	154(120-178)	162(140-177)	158(129-177.75)	0.719^§^
Intragroup p-value	0.798^¶^	0.882^¶^	-	-
Pre-creatinine (mg/dL)	7.23 ± 2.73	7.16 ± 2.3	7.19 ± 2.5	0.926^‡^
Post-creatinine (mg/dL)	8.14 ± 1.59	7.97 ± 1.34	8.05 ± 1.46	0.681^‡^
Intragroup p-value	0.036^**^	0.064^**^	-	-

Baseline hs-CRP and IL-6 were not significantly different between the two groups. Post-dialysis levels of IL-6 and hs-CRP increased significantly in both HD and CAPD groups, with intragroup p-values being 0.0003 and 0.0001 for IL-6 in the CAPD and HD groups, respectively, and <0.0001 for hs-CRP in both CAPD and HD groups (Table 4). The post-dialysis levels of IL-6 were not significantly different between the two groups ( p-value of 0.256 ); however, hs-CRP levels significantly increased in the HD group as compared to the CAPD group in the post-dialysis phase( p-value of 0.036).

**Table 4:** Comparison of inflammatory markers between CAPD and HD ^‡^Independent t-test; ^§^Mann-Whitney U-test; ^¶^Wilcoxon signed-rank test; **paired t-test CAPD: Continuous ambulatory peritoneal dialysis; HD: hemodialysis; IL-6: interleukin-6; hs-CRP: high-sensitivity C-reactive protein IL-6 normal value: <5(pg/ml); hs-CRP normal value: <5000(µg/L) The quantitative data having normal distribution is presented as the means ± SD and the nonnormal distributed data as median with 25th and 75th percentiles (interquartile range). A p-value of less than 0.05 was considered statistically significant. Post-dialysis levels of hs-CRP increased significantly in HD as compared to CAPD group with p-value of 0.036

Inflammatory markers	CAPD (n = 25)	HD (n = 25)	Total	p-value
Pre-IL-6 (pg/mL)	18.5 (16.54-24.67)	17.53 (16.39-28.58)	18.12 (16.428-26.245)	0.969^§^
Post-IL-6 (pg/mL)	44.44 (38.18-55.8)	46.56 (34.75-73.87)	44.86 (36.225-61.152)	0.256^§^
Intragroup p-value	0.0003^¶^	0.0001^¶^	-	-
Pre-hs-CRP (µg/L)	6274.36 ± 2141.81	6757.28 ± 1747.08	6515.82 ± 1949.71	0.387^‡^
Post-hs-CRP (µg/L)	10448.36 ± 1488.15	12377.94 ± 4133.76	11413.15 ± 3225.55	0.036^‡^
Intragroup p-value	<0.0001^**^	<0.0001^**^	-	-

Both post-dialysis BMI (intragroup p-value of <0.0001 for both HD and CAPD) and post-dialysis serum albumin (intragroup p-value of 0.0002 and <0.0001 for CAPD and HD group, respectively) significantly decreased in both the groups (Table 2, Table 5). However, on comparison between the two groups, the results were not statistically different in patients who underwent HD rather than CAPD (p-values of 0.388 and 0.132 for post-dialysis BMI and post-dialysis albumin group, respectively) (Table 2, Table 5).

**Table 5:** Comparison of body mass index (kg/m²) between CAPD and HD ^‡^Independent t-test; **paired t-test CAPD: Continuous ambulatory peritoneal dialysis; HD: hemodialysis Body mass index range: 18.5-24.9 (kg/m²) The quantitative data having normal distribution is presented as the mean ± SD. A p-value of less than 0.05 was considered statistically significant. BMI decreased significantly in both the groups post-dialysis (intragroup p-value of <0.0001 for both HD and CAPD)

Body mass index (kg/m²)	CAPD (n = 25)	HD (n = 25)	Total	p-value
Pre-body mass index (kg/m²)	23.83 ± 3.22	23.01 ± 3	23.42 ± 3.11	0.36^‡^
Post-body mass index (kg/m²)	22.72 ± 3.01	22 ± 2.84	22.36 ± 2.91	0.388^‡^
Intragroup p-value	<0.0001^**^	<0.0001^**^	-	-

**Figure 2:**
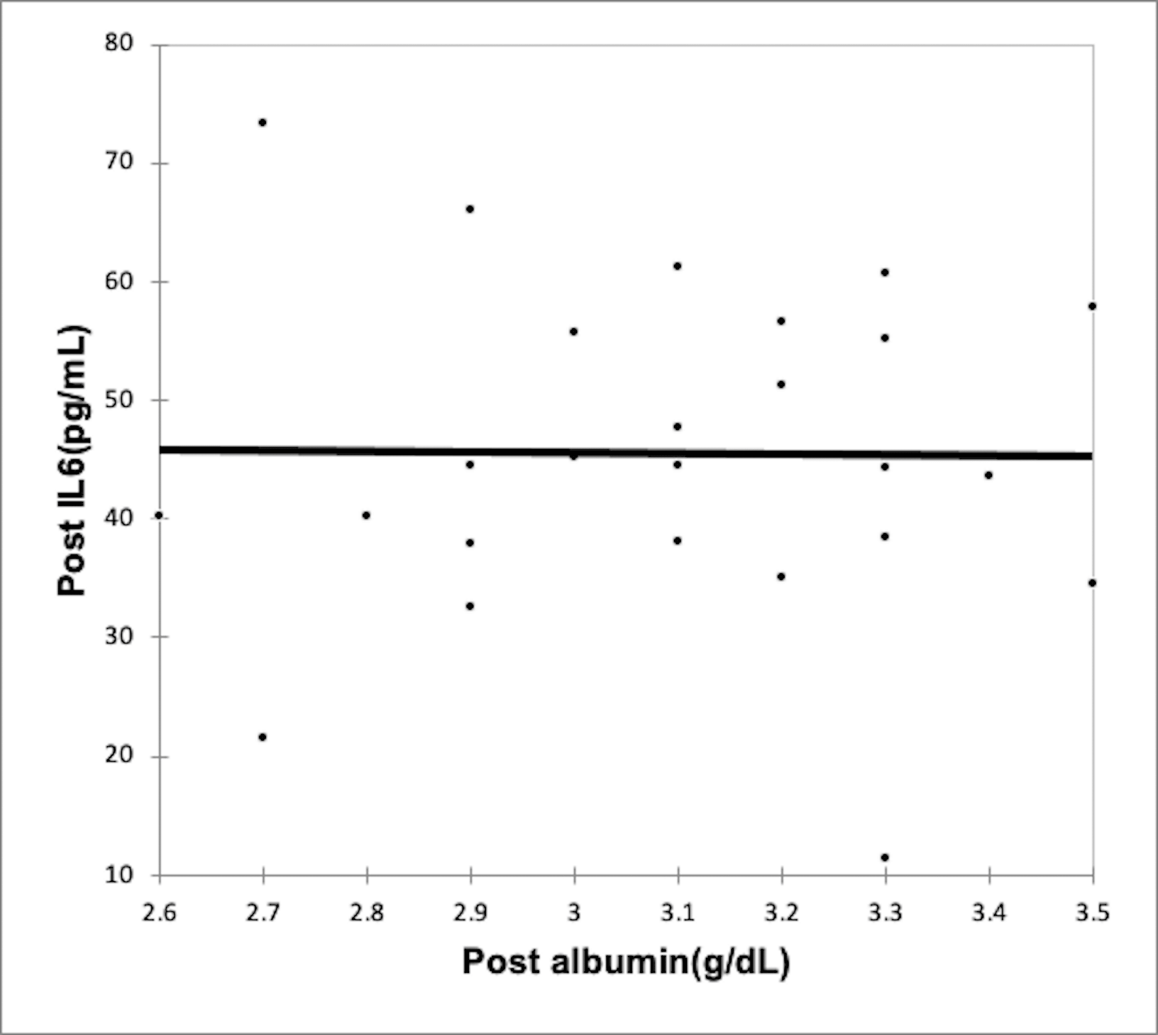
Correlation of post-dialysis albumin (g/dl) with post-dialysis Il-6 (pg/ml) in CAPD group CAPD: Continuous ambulatory peritoneal dialysis; IL-6: interleukin-6

**Figure 3:**
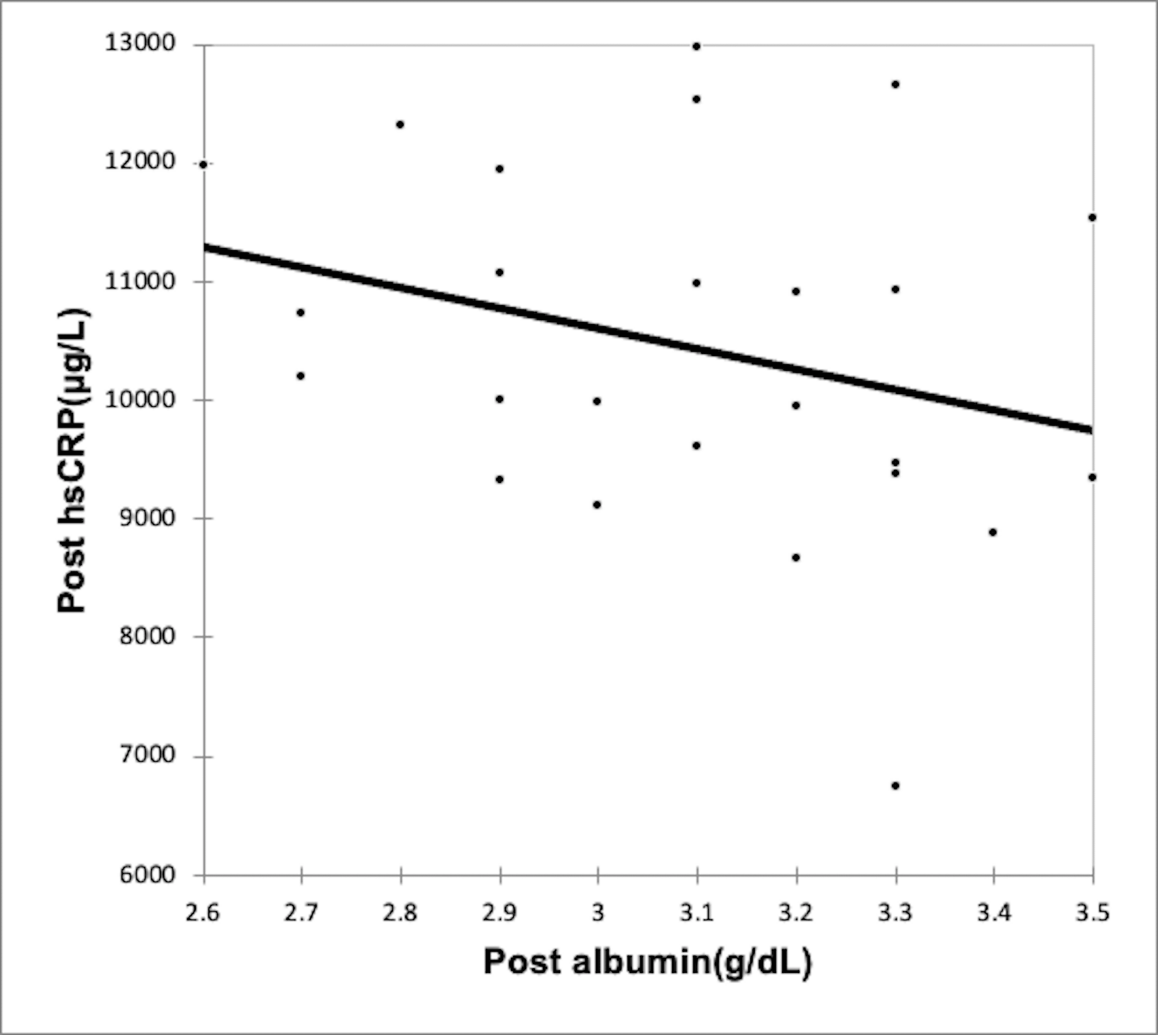
Correlation of post-dialysis albumin (g/dl) with post-dialysis hs-CRP (µg/L) in CAPD group CAPD: Continuous ambulatory peritoneal dialysis; hs-CRP: high-sensitivity C-reactive protein

**Figure 4:**
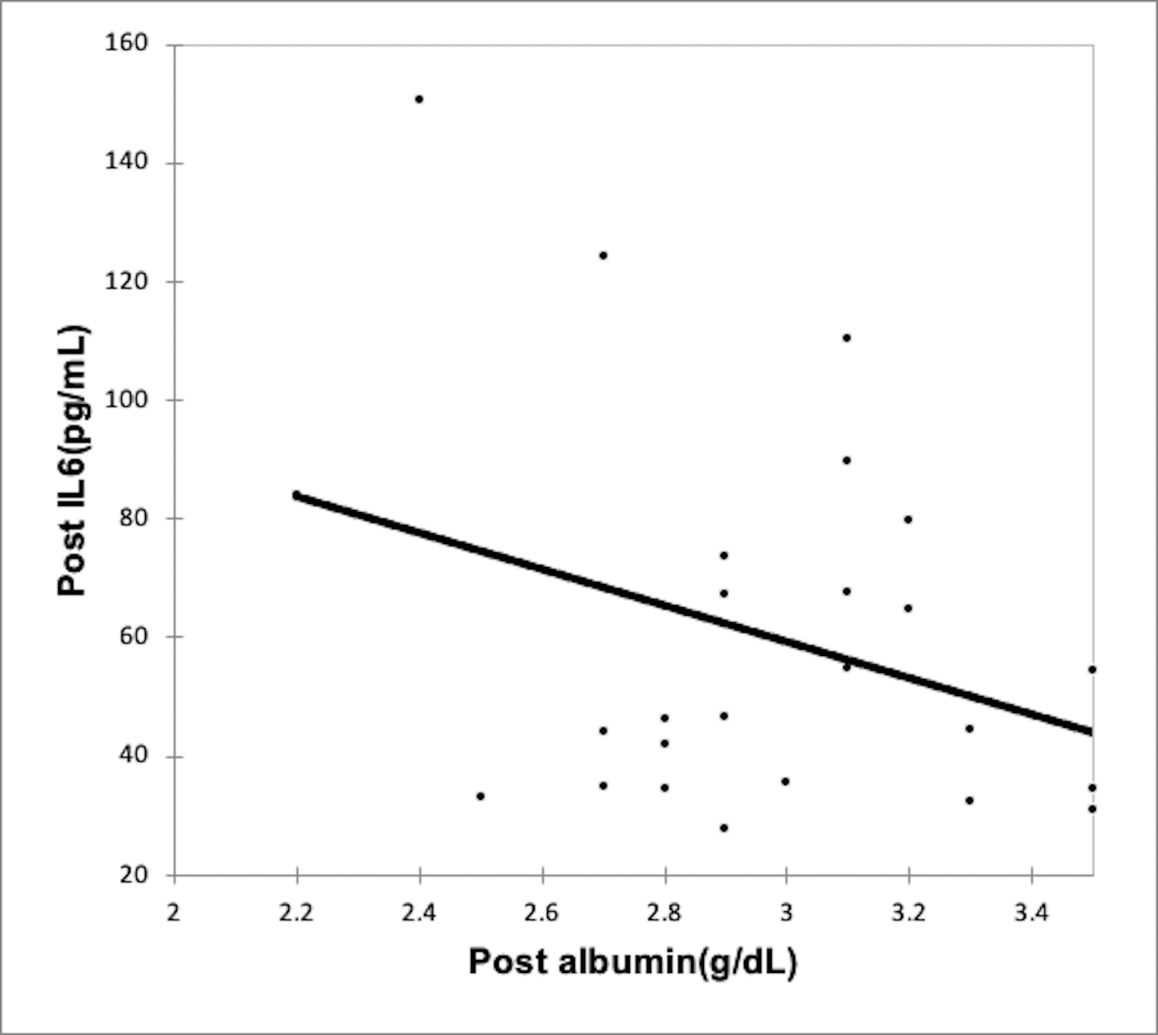
Correlation of post-dialysis albumin (g/dl) with post-dialysis Il-6 (pg/ml) in HD group HD: Hemodialysis; IL-6: interleukin-6

**Figure 5:**
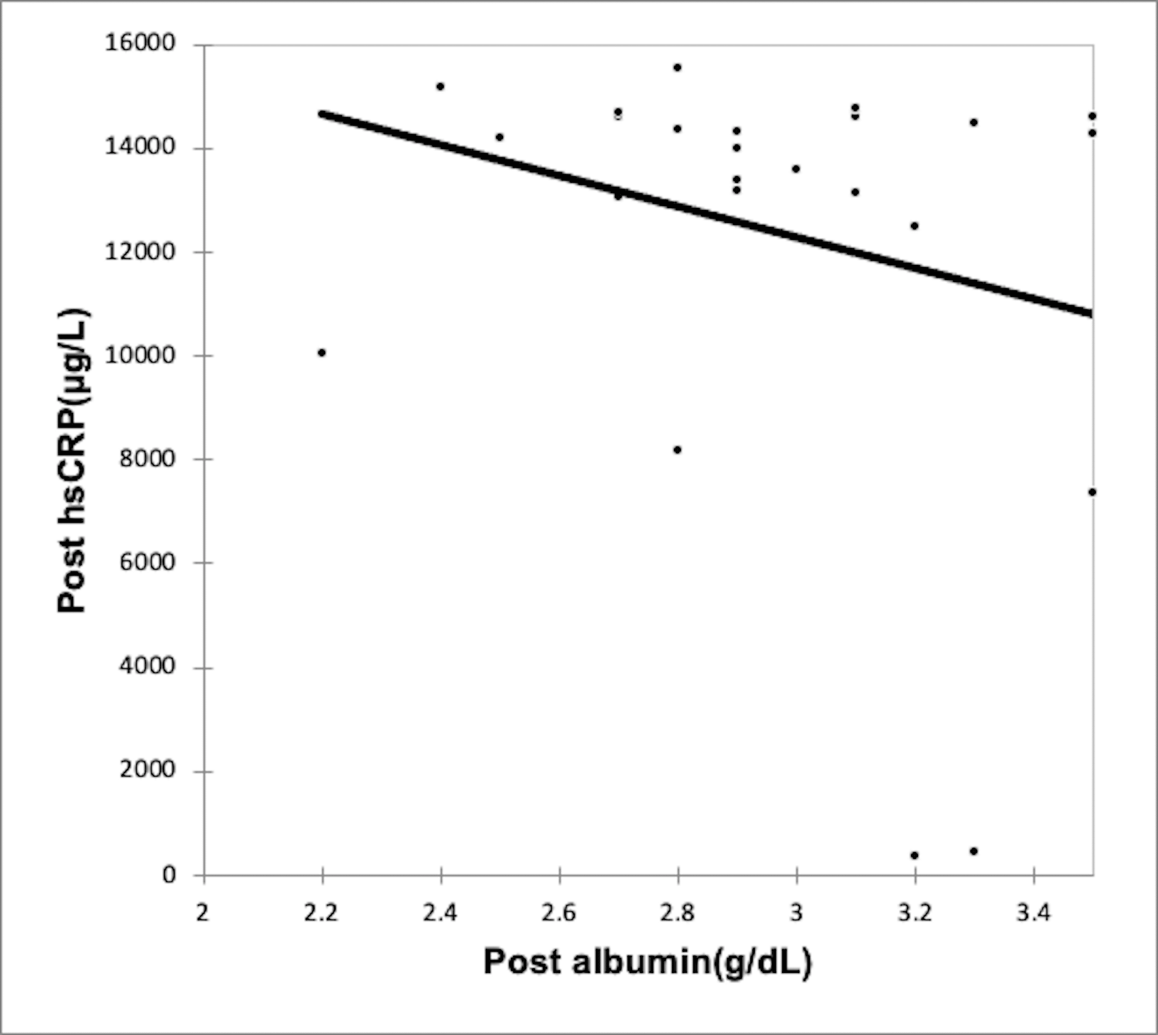
Correlation of post-dialysis albumin (g/dl) with post-dialysis hs-CRP (µg/L) in HD group HD: Hemodialysis; hs-CRP: high-sensitivity C-reactive protein

Using the Spearman rank correlation coefficient, we recognized a negative correlation between rising inflammation and serum albumin levels in both CAPD group (Figures 2-3, Table 6) and the HD group (Figures 4-5, Table 7); however, the correlation was not strong enough, with the p-value being nonsignificant.

**Table 6:** Correlation of post-dialysis albumin, IL-6, and hs-CRP with each other in CAPD group Spearman rank correlation coefficient CAPD: Continuous ambulatory peritoneal dialysis; IL-6: interleukin-6; hs-CRP: high-sensitivity C-reactive protein. A p-value of less than 0.05 was considered statistically significant

Variables	Post-albumin (g/dL)	Post-IL-6 (pg/mL)	Post-hs-CRP (µg/dL)
Post-albumin (g/dL)			
Correlation coefficient	-	0.009	-0.303
p-value	-	0.966	0.141
Post-IL-6 (pg/mL)			
Correlation coefficient	0.009	-	-0.216
p-value	0.966	-	0.298
Post-hs-CRP (µg/L)			
Correlation coefficient	-0.303	-0.216	-
p-value	0.141	0.298	-

**Table 7:** Correlation of post-dialysis albumin, IL-6, and hs-CRP with each other in HD group Spearman rank correlation coefficient HD: Hemodialysis; IL-6: interleukin-6; hs-CRP: high-sensitivity C-reactive protein A p-value of less than 0.05 was considered statistically significant

Variables	Post-albumin (g/dL)	Post-IL-6 (pg/mL)	Post-hs-CRP (µg/dL)
Post-albumin (g/dL)			
Correlation coefficient	-	-0.186	-0.214
p-value	-	0.372	0.302
Post-IL-6 (pg/mL)			
Correlation coefficient	-0.186	-	0.198
p-value	0.372	-	0.342
Post-hs-CRP (µg/L)			
Correlation coefficient	-0.214	0.198	-
p-value	0.302	0.342	-

In the CAPD group, we found hypoalbuminemia negatively correlated with increased hs-CRP only. In the HD group, both rising IL-6 and hs-CRP were negatively associated with a fall in albumin. We observed no significant difference in levels of post-dialysis serum albumin and inflammatory markers in diabetic and nondiabetic patients in both groups (Tables 8-10).

**Table 8:** Comparison of post-dialysis albumin (g/dL) and inflammatory markers between diabetics and nondiabetics in HD ^‡^Independent t-test; ^§^Mann-Whitney U-test HD: Hemodialysis; IL-6: interleukin-6; hs-CRP: high-sensitivity C-reactive protein The quantitative data having normal distribution is presented as the mean ± SD and the nonnormal distributed data as median with 25th and 75th percentiles (interquartile range). A p-value of less than 0.05 was considered statistically significant

Inflammatory markers	Diabetics (n = 3)	Nondiabetics (n = 22)	Total	p-value
Post-IL-6 (pg/mL)	31.04 (29.445-42.895)	50.45 (37.275-78.385)	46.56 (34.75-73.87)	0.079^§^
Post-hs-CRP (µg/L)	14560.67 ± 220.93	12080.3 ± 4330.25	12377.94 ± 4133.76	0.34^‡^
Post-albumin (g/dL)	Diabetics (n = 3)	Nondiabetics (n = 22)	Total	p-value
Mean ± SD	3.17 ± 0.31	2.94 ± 0.34	2.96 ± 0.34	0.273^‡^

**Table 9:** Comparison of post-dialysis albumin (g/dL) and inflammatory markers between diabetics and nondiabetics in CAPD ^‡^Independent t-test; ^§^Mann-Whitney U-test CAPD: Continuous ambulatory peritoneal dialysis; IL-6: interleukin-6; hs-CRP: high-sensitivity C-reactive protein The quantitative data having normal distribution is presented as the mean ± SD and the nonnormal distributed data as median with 25th and 75th percentiles (interquartile range). A p-value of less than 0.05 was considered statistically significant

Inflammatory markers	Diabetics (n = 11)	Nondiabetics (n = 14)	Total	p-value
Post-IL-6 (pg/mL)	40.18 (36.29-56.85)	44.48 (39.702-53.235)	44.44 (38.18-55.8)	0.743^§^
Post-hs-CRP (µg/L)	10625.9 ± 1389.84	10308.86 ± 1598.43	10448.36 ± 1488.15	0.608^‡^
Post-albumin (g/dL)	Diabetics (n = 11)	Nondiabetics (n = 14)	Total	p-value
Mean ± SD	3.06 ± 0.31	3.11 ± 0.19	3.09 ± 0.25	0.644^‡^

**Table 10:** Comparison of post-dialysis albumin (g/dL) and inflammatory markers between diabetics and nondiabetics in total study subjects ^‡^Independent t-test; ^§^Mann-Whitney U-test; IL-6: interleukin-6; hs-CRP: high-sensitivity C-reactive protein The quantitative data having normal distribution is presented as the mean ± SD and the nonnormal distributed data as median with 25th and 75th percentiles (interquartile range). A p-value of less than 0.05 was considered statistically significant

Inflammatory markers	Diabetics (n = 14)	Nondiabetics (n = 36)	Total	p-value
Post-IL-6 (pg/mL)	40.17 (33.1-55.537)	45.77 (38.322-66.425)	44.86 (36.225-61.152)	0.115^§^
Post-hs-CRP (µg/L)	11469.07 ± 2073.8	11391.41 ± 3600.93	11413.15 ± 3225.55	0.94^‡^
Post-albumin (g/dL)	Diabetics (n = 14)	Nondiabetics (n = 36)	Total	p-value
Mean ± SD	3.09 ± 0.3	3.01 ± 0.3	3.03 ± 0.3	0.4^‡^

## Discussion

CKD is a pro-inflammatory state, and dialysis adds to this inflammation; however, no precise data suggests which form of dialysis has more of an inflammatory response. Previous registry studies have shown a survival advantage with CAPD compared with HD; however, the mechanisms behind this disparity remain questionable [11,12].

We conducted this prospective observational study in a tertiary center in Kashmir Valley in India to assess the prevalence of inflammation in pre-dialysis and post-dialysis CKD patients with pre-dialysis states serving as internal controls. To the best of our knowledge, this study is the first in India to compare the inflammatory status within these two dialysis modalities. CRP and IL-6 levels were found to be increased in CKD patients in the pre-dialysis phase. Our results were consistent with other studies that found CRP and IL-6 levels to be elevated in pre-dialysis patients, which can contribute to worsening renal function and inflammation in these patients [5,9]. Both groups had significantly increased hs-CRP and IL-6 levels in the post-dialysis stage compared to the pre-dialysis stage. Our findings of increased hs-CRP [4] and IL-6 levels [8,13] in post-dialysis patients agree with others who have found these markers elevated in these patients. Comparing the two groups, interestingly, we observed a significant increase in hs-CRP levels in the HD group compared to the CAPD group (p-value < 0.05). There was no significant difference in post-dialysis IL-6 levels within the two groups. Some studies, like Haubitz et al. [14], stated that HD was associated with a more inflammatory response in elevated CRP than CAPD. In contrast, like the Filiopolous study [15], Borazan et al. also found no difference in the inflammatory markers in these two groups [16]. A recent study by Yong et al. inferred that HD was associated with significantly increased hs-CRP levels compared to PD [17].

Various dialysis-specific factors by which the HD stimulates inflammation include bio-incompatibility to the dialysis membrane, backfiltration of bacterial cell wall components, central venous/catheter-related infections, and contamination of dialysate solution with endotoxin, all of which can cause complement stimulation, monocyte activation, and upregulation of inflammatory cytokines and subsequently CRP [3,14]. Many HD-related factors, such as volume overload and biocompatibility of membranes, are diminished with PD. However, CAPD also has inflammation-specific associations like peritonitis, catheter site infection, and peritoneal fluid bio-incompatibility [18]. In addition, CAPD can lead to glucose absorption from the fluid, leading to increased oxidative stress and inflammation [19]. In our study, serum albumin was on the lower side in the pre-dialysis stage. Furthermore, mean albumin levels were significantly diminished in both the groups in the dialysis phase compared to the pre-dialysis phase. Mean albumin levels were marginally higher in the CAPD group than in the HD group, but the result was insignificant. This contrasts with the study that shows serum albumin is lower in the PD group by 0.3 gm/dl than in the HD group due to renal albumin losses during peritoneal dialysis [20].

The possible explanation of lower serum albumin in HD could be due to higher inflammation in the form of increased hs-CRP in HD as compared to CAPD, which could have led to more pronounced hypoalbuminemia in the HD group [21]. Also, we observed no significant difference in inflammatory markers and albumin levels in diabetic vs. nondiabetic post-dialysis patients in both groups, underlying the role of other mechanisms, such as the dialysis modality resulting in the inflammation. Multiple studies have shown a negative correlation between inflammatory markers and serum albumin levels. Some have shown IL-6 to be independently correlated with albumin and the stage of CKD [9], while in others, CRP was negatively correlated with serum albumin [21]. In our study, rising hs-CRP levels were shown to be negatively correlated with lower albumin in both groups; in contrast, rising IL-6 levels were shown to be negatively correlated to albumin in the HD group, only possibly indicating CRP-driven inflammation to be more pronounced cause of hypoalbuminemia.

It has been shown that reduced serum albumin in dialysis patients can be regarded as a sign of chronic inflammation rather than malnutrition [22], so the marked hypoalbuminemia in the HD group can be viewed as a sign of a more inflammatory burden in that group. However, the correlation between serum albumin and inflammatory markers was statistically insignificant in our study. Our limited sample size could have prevented us from dissecting a statistically significant correlation. BMI also decreased in the post-dialysis phase in HD and CAPD patients, but the differences between the two groups were not statistically significant. Inflammation may be a causative factor for malnutrition and reduced BMI in CKD patients [23].

CRP and IL-6 levels have been shown to predict mortality in both pre-and post-dialysis CKD patients. Elevated CRP [4] and IL-6 [7,24] have independently predicted mortality in HD patients. However, another study found that albumin rather than CRP was independently associated with mortality in this population group [25]. Studies on the association between elevated CRP and mortality in CAPD patients have not given consistent results [6,26]. Using risk prediction by multivariant model, Tripepi et al. [27] proved that IL-6 was a better predictor of cardiovascular mortality than CRP. A recent meta-analysis also demonstrated that IL-6 was more strongly associated with cardiovascular mortality than other markers, particularly in HD patients [28]. Another recent systematic review confirms the adverse association between IL-6 levels and survival in dialysis patients [29]. None of our patients died within the study period. The possible explanations could be the short study period and the inclusion of relatively healthy ESRD patients. Since our study found lesser inflammation in CAPD compared to HD, there could be a possible survival advantage with the initiation of CAPD; however, studies with larger sample sizes are essential for establishing this point.

This study's strengths are its prospective design, complete data collection, and maintenance of the initial dialysis modality balance at follow-up. The limitations of this study include a lack of randomization, which can lead to selection bias, as there is an increased proportion of diabetics in CAPD group. The study was based on a single-time assessment of inflammatory markers at the pre-and post-dialysis phases. Our results were obtained from relatively healthy CKD patients and do not represent every cohort of ESRD patients .

## Conclusions

CKD is associated with a chronic inflammatory state. Markers of inflammation like IL-6 and hs-CRP are elevated in ESRD patients irrespective of etiology and are shown to be strong prognosticators of poor outcomes. Cardiovascular mortality is increased in such patients which is not explained by traditional risk factors, thereby implying nonconventional risk factors like dialysis-related factors resulting in higher inflammatory burden. This study was done to identify the effect of dialysis modality on inflammatory markers and to determine which dialysis modality has a more inflammatory burden on the patient. In our study, ESRD patients manifest a more significant inflammatory response with the institution of HD compared with CAPD. Inflammation-associated hypoalbuminemia was more predominant in HD patients, and albumin concentration is negatively correlated with established markers of inflammation. There could be a possible survival advantage with the initiation of CAPD compared to HD; however, studies with a larger cohort are required to prove this point.

## References

[1] Stenvinkel P, Alvestrand A (2002). Inflammation in end-stage renal disease: sources, consequences, and therapy. Semin Dial.

[2] Cheung AK, Sarnak MJ, Yan G (2000). Atherosclerotic cardiovascular disease risks in chronic hemodialysis patients. Kidney Int.

[3] Amore A, Coppo R (2002). Immunological basis of inflammation in dialysis. Nephrol Dial Transplant.

[4] Yeun JY, Levine RA, Mantadilok V (2000). C-reactive protein predicts all-cause and cardiovascular mortality in hemodialysis patients. American journal of kidney diseases.

[5] Ortega O, Rodriguez I, Gallar P (2002). Significance of high C-reactive protein levels in pre-dialysis patients. Nephrol Dial Transplant.

[6] Noh H, Lee SW, Kang SW (1998). Serum C-reactive protein: a predictor of mortality in continuous ambulatory peritoneal dialysis patients. Perit Dial Int.

[7] Rao M, Guo D, Perianayagam MC, Tighiouart H, Jaber BL, Pereira BJ, Balakrishnan VS (2005). Plasma interleukin-6 predicts cardiovascular mortality in hemodialysis patients. Am J Kidney Dis.

[8] Pecoits-Filho R, Bárány P, Lindholm B, Heimbürger O, Stenvinkel P (2002). Interleukin-6 is an independent predictor of mortality in patients starting dialysis treatment. Nephrol Dial Transplant.

[9] Barreto DV, Barreto FC, Liabeuf S (2010). Plasma interleukin-6 is independently associated with mortality in both hemodialysis and pre-dialysis patients with chronic kidney disease. Kidney Int.

[10] Agarwal SK, Srivastava RK (2009). Chronic kidney disease in India: challenges and solutions. Nephron Clin Pract.

[11] McDonald SP, Marshall MR, Johnson DW, Polkinghorne KR (2009). Relationship between dialysis modality and mortality. J Am Soc Nephrol.

[12] Vonesh EF, Snyder JJ, Foley RN, Collins AJ (2006). Mortality studies comparing peritoneal dialysis and hemodialysis: what do they tell us?. Kidney Int Suppl.

[13] Kato A, Odamaki M, Takita T, Maruyama Y, Kumagai H, Hishida A (2002). Association between interleukin-6 and carotid atherosclerosis in hemodialysis patients. Kidney Int.

[14] Haubitz M, Brunkhorst R, Wrenger E, Froese P, Schulze M, Koch KM (1996). Chronic induction of C-reactive protein by hemodialysis, but not by peritoneal dialysis therapy. Perit Dial Int.

[15] Filiopoulos V, Hadjiyannakos D, Takouli L, Metaxaki P, Sideris V, Vlassopoulos D (2009). Inflammation and oxidative stress in end-stage renal disease patients treated with hemodialysis or peritoneal dialysis. Int J Artif Organs.

[16] Borazan A, Ustün H, Ustundag Y, Aydemir S, Bayraktaroglu T, Sert M, Yilmaz A (2004). The effects of peritoneal dialysis and hemodialysis on serum tumor necrosis factor-alpha, interleukin-6, interleukin-10 and C-reactive-protein levels. Mediators Inflamm.

[17] Yong K, Dogra G, Boudville N, Lim W (2018). Increased inflammatory response in association with the initiation of hemodialysis compared with peritoneal dialysis in a prospective study of end-stage kidney disease patients. Perit Dial Int.

[18] Pecoits-Filho R, Stenvinkel P, Wang AY, Heimbürger O, Lindholm B (2004). Chronic inflammation in peritoneal dialysis: the search for the holy grail?. Perit Dial Int.

[19] Fortes PC, Versari PH, Stinghen AE, Pecoits-Filho R (2007). Controlling inflammation in peritoneal dialysis: the role of pd-related factors as potential intervention targets. Perit Dial Int.

[20] Goldwasser P, Feldman JG, Barth RH (2002). Serum prealbumin is higher in peritoneal dialysis than in hemodialysis: a meta-analysis. Kidney Int.

[21] Alves FC, Sun J, Qureshi AR (2018). The higher mortality associated with low serum albumin is dependent on systemic inflammation in end-stage kidney disease. PLoS One.

[22] Rippe B, Öberg CM (2016). Albumin turnover in peritoneal and hemodialysis. Semin Dial.

[23] Graterol Torres F, Molina M, Soler-Majoral J (2022). Evolving concepts on inflammatory biomarkers and malnutrition in chronic kidney disease. Nutrients.

[24] Beberashvili I, Sinuani I, Azar A (2011). IL-6 levels, nutritional status, and mortality in prevalent hemodialysis patients. Clin J Am Soc Nephrol.

[25] Owen WF, Lowrie EG (1998). C-reactive protein as an outcome predictor for maintenance hemodialysis patients. Kidney Int.

[26] Herzig KA, Purdie DM, Chang W (2001). Is C-reactive protein a useful predictor of outcome in peritoneal dialysis patients?. J Am Soc Nephrol.

[27] Tripepi G, Mallamaci F, Zoccali C (2005). Inflammation markers, adhesion molecules, and all-cause and cardiovascular mortality in patients with ESRD: searching for the best risk marker by multivariate modeling. J Am Soc Nephrol.

[28] Chen Z, Wang Y (2023). Interleukin-6 levels can be used to estimate cardiovascular and all-cause mortality risk in dialysis patients: a meta-analysis and a systematic review. Immun Inflamm Dis.

[29] Istanbuly O, Belcher J, Tabinor M, Solis-Trapala I, Lambie M, Davies SJ (2023). Estimating the association between systemic Interleukin-6 and mortality in the dialysis population. Re-analysis of the global fluid study, systematic review and meta-analysis. BMC Nephrol.

